# Primary thromboprophylaxis in ambulatory symptomatic patients with COVID-19: a systematic review and meta-analysis of randomized controlled trials

**DOI:** 10.1016/j.rpth.2024.102613

**Published:** 2024-10-29

**Authors:** Davide Di Vece, Marco Valgimigli, Elliot Barnathan, Jean M. Connors, Frank Cools, Ulrike Held, Ajay K. Kakkar, Gregory Piazza, David Spirk, Saverio Virdone, Nils Kucher, Stefano Barco

**Affiliations:** 1 Department of Internal Medicine B, University Medicine Greifswald, Greifswald, Germany; 2 First Clinic of Internal Medicine, Department of Internal Medicine, University of Genoa, Genoa, Italy; 3 Department of Cardiology, University Hospital Zurich, Zurich, Switzerland; 4 Cardiocentro Ticino Institute, Ente Ospedaliero Cantonale (EOC), Lugano, Switzerland; 5 Johnson and Johnson, Raritan, New Jersey, USA; 6 Hematology Division, Brigham and Women’s Hospital, Harvard Medical School, Boston, Massachusetts, USA; 7 Department of Cardiology, General Hospital Klina, Brasschaat, Belgium; 8 Department of Biostatistics at Epidemiology, Biostatistics and Prevention Institute, University of Zurich, Zurich, Switzerland; 9 Thrombosis Research Institute, London, United Kingdom; 10 Division of Cardiovascular Medicine, Department of Medicine, Brigham and Women’s Hospital, Harvard Medical School, Boston, Massachusetts, USA; 11 Institute of Pharmacology, University of Bern, Bern, Switzerland; 12 Department of Angiology, University Hospital Zurich, Zurich, Switzerland; 13 Center for Thrombosis and Hemostasis, University Medical Center of the Johannes Gutenberg University Mainz, Mainz, Germany

**Keywords:** COVID-19, thromboprophylaxis, venous thromboembolism, outpatients

## Abstract

**Background:**

The global impact of the COVID-19 pandemic has prompted the search for strategies to improve outcomes in affected individuals, including those initially managed in outpatient settings. Thromboembolic events have been reported as a concerning complication.

**Objectives:**

The aim of this study was to evaluate efficacy and safety of primary thromboprophylaxis in outpatients with COVID-19. The study protocol was registered in PROSPERO (CRD42022362776).

**Methods:**

The study adhered to Preferred Reporting Items for Systematic Reviews and Meta-Analyses guidelines and conducted a comprehensive search of PubMed/MEDLINE, ClinicalTrials.gov, and OVID/Embase and CENTRAL from Cochrane for studies up to December 11, 2023, without language restrictions. Randomized controlled trials comparing prophylactic-dose anticoagulation with placebo or standard of care in symptomatic outpatients with COVID-19 were included in this analysis. The primary outcome was the composite of all-cause hospitalization and death within 30 days. Secondary outcomes included venous thromboembolism, the composite of venous thromboembolism and major arterial cardiovascular events, and the individual components of the primary outcome.

**Results:**

Seven randomized controlled trials and 3758 COVID-19 outpatients were included. When compared with placebo or standard of care, thromboprophylaxis was associated with similar rates of all-cause hospitalization or mortality (relative risk, 1.00; 95% CI, 0.77-1.31) and lower rates of venous thromboembolism (relative risk, 0.28; 95% CI, 0.08-0.94), corresponding to a 0.6% absolute risk reduction and number needed to treat of 174.

**Conclusion:**

Thromboprophylaxis in symptomatic COVID-19 outpatients led to reduction in venous thromboembolism risk, with no impact on hospitalization or death. However, the overall low absolute risk reduction may not support its routine use.

## Essentials

• Thromboembolic events have been reported as possible complications in COVID-19 patients.
• This study assessed thromboprophylaxis in symptomatic outpatients with COVID-19.
• Thromboprophylaxis modestly reduced venous thromboembolism risk but not death or hospitalization.
• Low absolute benefit suggests that routine thromboprophylaxis in outpatients is not warranted.

## 1 Introduction

Venous and arterial thromboembolic events are well-described complications of COVID-19 and are associated with worse prognosis [1,2]. Therapeutic-dosed heparin improved outcomes in selected, moderately ill hospitalized COVID-19 patients, as compared with a standard prophylactic regimen [3]. The use of primary thromboprophylaxis in patients initially managed in the ambulatory setting is not supported by firm evidence.

Available randomized controlled trials have yielded negative or inconclusive results. These included studies comparing different thromboprophylaxis agents with control/placebo among outpatients with COVID-19 [[4], [5], [6], [7], [8], [9], [10]]. The relatively low sample size in individual studies may partly explain these results. A recent meta-analysis focusing on anticoagulant treatments as well as on antiplatelet agents was published before the completion of all trials and led to similar conclusions, namely that low-certainty evidence indicates that thromboprophylaxis may result in little or no difference in adverse events among outpatients with COVID-19 [11].

The optimal approach for anticoagulation therapy in ambulatory patients with COVID-19 remains unknown. The aim of this systematic review and meta-analysis of randomized controlled trials is to assess the efficacy and safety of primary thromboprophylaxis in symptomatic outpatients with COVID-19.

## 2 Methods

The Preferred Reporting Items for Systematic Reviews and Meta-Analyses reporting guidelines were followed. Studies were screened without language restrictions in PubMed/MEDLINE, ClinicalTrials.gov, OVID/Embase and CENTRAL from Cochrane up to December 11, 2023. In addition, presentations at international cardiovascular conferences were searched. The study protocol was registered in PROSPERO (CRD42022362776). The investigators of all individual trials were contacted to obtain additional data or queried about specific endpoints.

Randomized controlled trials were considered eligible if they included adult symptomatic subjects with COVID-19 who were initially managed in the ambulatory setting and had no other indications for anticoagulation. Studies including patients that commenced thromboprophylaxis after hospital discharge were not included. Subjects were randomized to receive either prophylactic-dosed anticoagulation or placebo/standard of care (no additional antithrombotic therapy).

The primary outcome of the study was a composite of all-cause hospitalization and death within 30 days after randomization. The secondary efficacy outcomes were (i) objectively-diagnosed venous thromboembolism (VTE) accounting for deep vein thrombosis and pulmonary embolism, as defined in the individual studies; (ii) a composite of VTE and major arterial cardiovascular events, as defined in the individual trials; and (iii) the individual components of the primary outcome, all within 30 days after randomization. The primary safety outcome was major bleeding (International Society on Thrombosis and Haemostasis criteria or similarly defined) [12]. For studies with a follow-up period of <30 days, the necessary data were obtained directly from the principal investigators.

Study screening and selection and data extraction were independently conducted by 2 authors (D.D.V., S.B.). The Preferred Reporting Items for Systematic Reviews and Meta-Analyses flow chart and the overarching search strategy are available as Supplementary Material (Supplementary Figure S1, Supplementary Literature-search strategy). For quality assessment, the Cochrane risk of bias assessment tool version 2 was used, and the assessment was performed independently by 3 authors (D.D.V., D.S., S.B.). The risk of bias related to individual items was rated as low, high, or unclear for each trial (Supplementary Figures S2 and S3).

The primary outcome analysis was performed in the intention-to-treat populations as reported in individual studies. Risk estimates of the 30-day cumulative incidences were calculated from the original data. The relative risk (RR), risk difference, and number needed to treat (NNT) were used as effect measures: to pool the effect measures across studies, a random-effects model was applied to account for between-study heterogeneity. Heterogeneity was assessed with the heterogeneity variance parameter tau^2^ (estimated with restricted maximum likelihood) and Higgins-Thompsons’ *I*^2^ index. ReviewManager version 5.0 (Cochrane Collaboration) served for data analysis.

## Results and Discussion

We identified 7 randomized controlled trials matching the eligibility criteria, comprising 3758 outpatients with COVID-19 (flowchart of included studies available as Supplementary Figure S1). Overall, 1871 patients were randomized to receive thromboprophylaxis, whereas 1887 patients received placebo or standard of care without anticoagulation (Table 1). The inclusion and exclusion criteria for the studies included in the meta-analysis are detailed in the Supplementary Table S1, while the enrolment periods are provided in the Supplementary Table S2. Primary thromboprophylaxis consisted of rivaroxaban in 3 trials, enoxaparin in 2 trials, and apixaban in 2 trials. Three trials included a placebo comparator, and 4 were conducted as open-label studies. In individual studies, the median or mean age ranged between 44 and 61 years, and the proportion of women ranged from 44% to 61%. The prevalence of vaccinated patients was overall low (Table 2) [13]. A summary of the quality assessment of individual studies is available in Supplementary Figure S2.

**Table 1:** Characteristics of the studies included in the meta-analysis.

Study	Countries	Sample size (ITT)	Intervention	Comparator	Follow-up (d)	Primary outcome	Secondary outcomes
Connors et al. [4]	United States	271	Apixaban 2.5 mg twice a day for 45 d	Placebo	45	Composite of symptomatic VTE, PE, arterial thromboembolism, MI, ischemic stroke, hospitalization for cardiovascular or pulmonary events, all-cause death	Individual component of the primary outcome
Ananworanich et al. [5]	United States	444	Rivaroxaban 10 mg once a day for 21 d	Placebo	28	Progression to moderate or severe disease according to Gates MRI scale	Time to disease resolution, incidence of hospitalization, proportion of patients with disease progression, resolution, and distribution of Gates MRI and WHO scale scores
Barco et al. [6]	Switzerland, Germany	472	Enoxaparin 40 mg once a day for 14 d	Standard of care	30	Composite of hospitalization and all-cause death	Composite of cardiovascular events^b^; individual component of primary endpoint, DIC, net clinical benefit
Cools et al. [7]	Belgium, Brazil, India, South Africa, Spain, United Kingdom	219	Enoxaparin 40 mg once a day^a^ for 21 d	Standard of care	21	Composite of hospitalization and all-cause death	VTE, bleeding
Piazza et al. [8]	United States	1284	Rivaroxaban 10 mg once a day for 35 d	Standard of care	35	Composite of symptomatic VTE, MI, ischemic stroke, acute limb ischemia, CNS systemic arterial embolism, hospitalization, and all-cause death	Emergency department visits major, clinically relevant nonmajor bleeding
Avezum et al. [9]	Brazil	657	Rivaroxaban 10 mg once a day for 14 d	Standard of care	30	Composite of VTE, mechanical ventilation, MACE, death	Time to hospitalization, admission to ICU, need for orotracheal intubation, vascular endpoint I and II,^c^ major bleeding, death
de Barros et al. [10]	Brazil	411	Apixaban 2.5 mg twice a day for 30 d	Placebo	30	Composite of hospitalization and all-cause death	Individual component of the primary outcome, composite of arterial and venous thrombosis, bleeding

CNS, central nervous system; DIC, disseminated intravascular coagulation; ICU, intensive care unit; ITT, intention to treat; MACE, acute myocardial infarction, stroke, or acute limb ischemia; MI, myocardial infarction; MRI, Medical Research Institute; PE, pulmonary embolism; VTE, venous thromboembolism; WHO, World Health Organization.

a Patients who weighed ≥100 kg received enoxaparin 40 mg twice a day.

b VTE, MI, or myocarditis; peripheral arterial ischemic events; acute splanchnic vein thrombosis; and ischemic stroke.

c Vascular endpoint I: nonfatal MI, nonfatal ischemic stroke, cardiovascular death, or VTE; vascular endpoint II: cardiovascular death, nonfatal MI, nonfatal ischemic stroke, acute limb ischemia, or VTE.

**Table 2:** Characteristics of the study population in the individual studies included in the meta-analysis.

Study	Age (y)	Women	Body mass index (kg/m^2^)	History of smoking	Arterial hypertension	Diabetes mellitus	Chronic lung disease	Chronic heart failure	Malignancy	Complete vaccination
Connors et al. [4]	54 (46-59)	191 (58)	30	60 (18)	120 (36)	60 (18)	Not available	Not available	Not available	0
Ananworanich et al. [5]	49 (18-83)	267 (60)	35 (17-69)	Not available	230 (51.8)	123 (27.7)	27 (6.1)	Not available	Not available	0
Barco et al. [6]	57 (53-62)	217 (46)	26 (5)	81 (17)	115 (24)	38 (8)	9 (2)	2 (<1)	22 (5)	36 (7.6)
Cools et al. [7]	59 (50-67)	96 (44)	30	59 (28)	114 (70)	50 (31)	20 (12)	1 (1)	2 (1)	0
Piazza et al. [8]	56 (13)	783 (61)	34 (14-67)	432 (34)	470 (36.6)	274 (21.3)	27 (2.1)	17 (1.3)	161 (12.5)	27 (2.1)
Avezum et al. [9]	61 (47-69)	365 (56)	31 (27-35)	78 (11.9)^a^	521 (79.3)	235 (35.8)	27 (4.1)	11 (1.7)	36 (5.5)	Not available
de Barros et al. [10]^b^	44 (14)	240 (58)	29	Not available	129 (31)	49 (12)	Not available	1 (<1)	Not available	313 (88.4)

Data are presented as n (% of available data or applicable groups in the case of multiple study arms), mean (SD), or median (Q1-Q3), unless otherwise specified.

Complete vaccination refers to the administration of ≥2 doses of vaccine.

a Defined as current smoking.

b Part of the data in this study were drawn from the data presented at an international cardiovascular conference (ESC Congress 2022) as they were not included in the recently published research letter [13].

**Figure 1:**
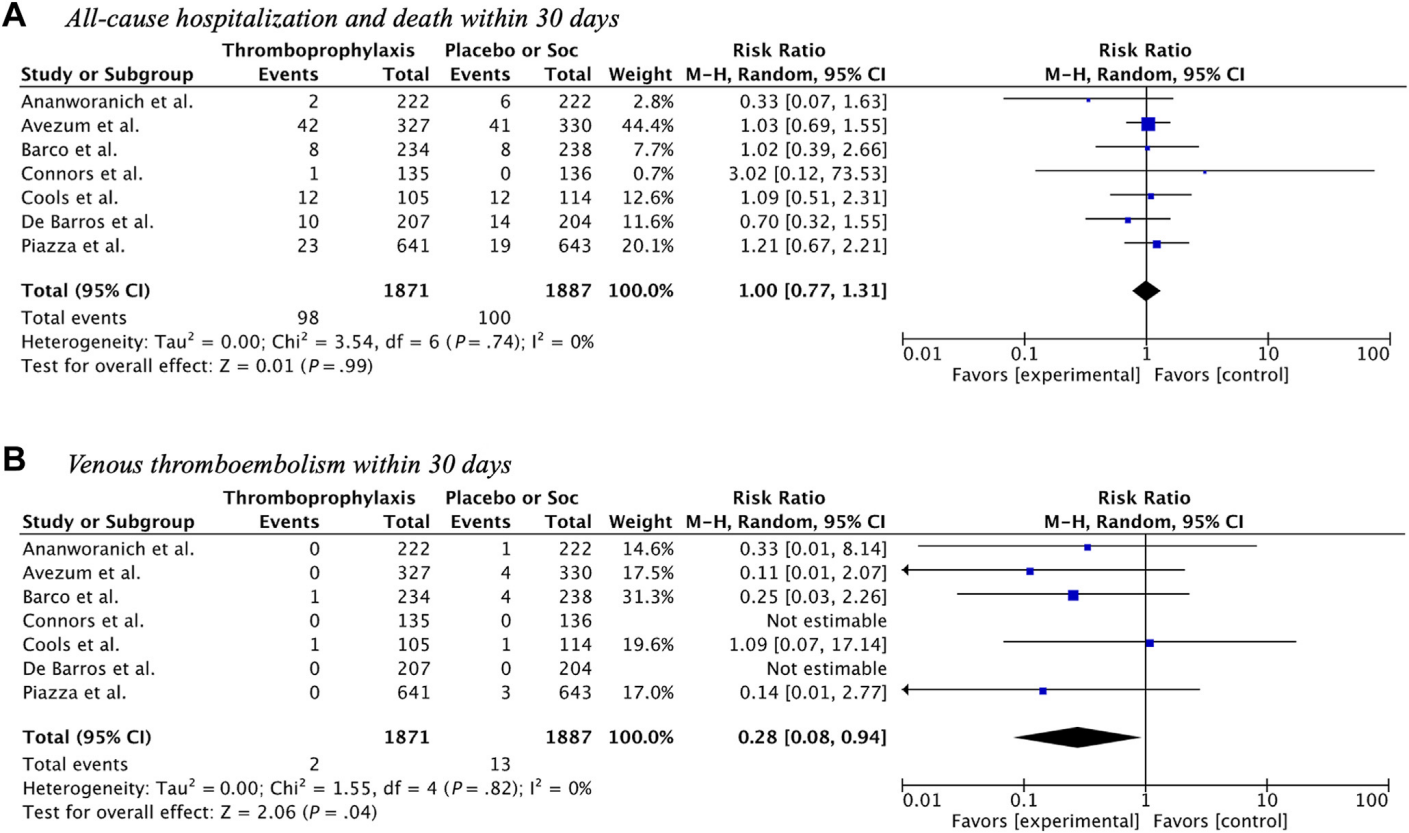
Primary and secondary outcome of the meta-analysis. M-H, Mantel–Haenszel test; Soc, standard of care.

A total of 198 patients experienced all-cause hospitalization or death within 30 days after randomization. A total of 98 (5.2%) events occurred in patients receiving thromboprophylaxis vs 100 (5.3%) events in the control group, resulting in a RR of 1.00 (95% CI, 0.77-1.31; tau^2^ = 0 and *I*^2^ = 0%; Figure 1A).

A total of 15 VTE events occurred within 30 days after randomization: of these, 2 (0.1%) occurred in patients receiving thromboprophylaxis vs 13 (0.7%) in the control group, corresponding to a RR of 0.28 (95% CI, 0.08-0.94; tau^2^ = 0 and *I*^2^ = 0%; Figure 1B). The NNT to prevent one case of VTE was 174 (95% CI, 103-567). A total of 30 patients experienced arterial or venous cardiovascular events within 30 days after randomization: 9 (0.5%) occurred in the thromboprophylaxis group vs 21 (1.1%) in the control group, resulting in a RR of 0.52 (95% CI, 0.24-1.12; tau^2^ = 0 and *I*^2^ = 0%; Supplementary Figure S3A).

Hospitalization from any cause occurred in 179 patients, of whom 90 (4.8%) were receiving thromboprophylaxis and 89 (4.7%) were not, resulting in a RR of 1.04 (95% CI, 0.78-1.38; tau^2^ = 0 and *I*^2^ = 0%; Supplementary Figure S3B). Death was recorded in 22 patients, of whom 9 (0.5%) were receiving thromboprophylaxis and 13 (0.7%) were not, corresponding to a RR of 0.72 (95% CI, 0.31-1.65; tau^2^ = 0 and *I*^2^ = 0%; Supplementary Figure S3C).

A total of 3 patients experienced major bleeding within 30 days after randomization: 2 (0.1%) occurred in patients receiving thromboprophylaxis vs 1 (0.1%) in controls for a RR of 1.48 (95% CI, 0.23-9.38; tau^2^ = 0.00; *I*^2^ = 0%).

The present analysis provides current evidence on primary thromboprophylaxis in symptomatic outpatients with COVID-19 initially managed in the ambulatory setting. To our knowledge, no further ongoing trials investigate this specific research question. Our results indicate that thromboprophylaxis did not influence the 30-day risk of all-cause hospitalization and death. However, it led to a 70% reduction in the risk of objectively verified VTE, corresponding to a 0.6% risk reduction in absolute terms and a NNT of 174. We found no difference between groups in the risk of all-cause mortality, need for hospitalization, the composite of arterial or venous events, and major bleeding.

No individual trial demonstrated a reduction of VTE among COVID-19 outpatients with thromboprophylaxis due to their limited power. The same applies to pooled analysis focusing on enoxaparin trials only [14]. In our meta-analysis, thromboprophylaxis resulted in a reduction in the risk of VTE at 30 days. Despite statistical significance at a population level, the overall low absolute risk among controls, approximately 0.7%, indicates that routine thromboprophylaxis in these patients may not be warranted. Furthermore, these results may also inform the approach to other groups of outpatients with respiratory and infectious disorders characterized by increased thromboembolic risk. In addition, we showed that the use of thromboprophylaxis did not increase the risk of major bleeding.

Limitations of the present study need to be acknowledged. First, the studies were heterogeneous in terms of demographic and clinical characteristics of the included study population, timeframe and virus variants, geographic setting, and prevalence of vaccination. Second, different types of prophylactic-dosed anticoagulants including oral and subcutaneous drugs were used across studies. Third, data on clinically relevant nonmajor bleeding events were not available in most studies, and major bleeding events were not externally adjudicated in all trials. Furthermore, detailed data on arterial events were predominantly available only as part of composite outcomes, precluding separate analyses. Finally, the low numbers of outcome events did not allow sensitivity analyses or subgroup analyses focusing on specific risk factors.

In conclusion, thromboprophylaxis in symptomatic COVID-19 outpatients resulted in a modest yet statistically significant reduction in VTE risk, with no impact on all-cause hospitalization or death. Given the limited absolute risk reduction observed, these findings do not justify the routine use of thromboprophylaxis at a broad population level.
