# Supplementary material for: Primary thromboprophylaxis in ambulatory symptomatic patients with COVID-19: a systematic review and meta-analysis of randomized controlled trials
Source: Res Pract Thromb Haemost. 2024 Oct 29;8(8):102613. doi: 10.1016/j.rpth.2024.102613 (PMC11652863; doi:10.1016/j.rpth.2024.102613)
Supplement: Supplementary Material [file mmc1.docx]

**SUPPLEMENTARY MATERIAL**

**Primary thromboprophylaxis in ambulatory symptomatic patients with COVID-19: A systematic review and meta-analysis of randomized controlled trials**

Davide Di Vece^1,2,3^, MD; Marco Valgimigli^4^, MD, PhD; Elliot Barnathan^5^, Jean M. Connors^6^, MD; Frank Cools^7^, MD; Ulrike Held^8^, PhD; Ajay K. Kakkar^9^, MD, PhD; Gregory Piazza^10^, MD; David Spirk^11^, MD; Saverio Virdone^9^, MSc; Nils Kucher^12^, MD; Stefano Barco^12,13^, MD, PhD

1. Department of Internal Medicine B, University Medicine Greifswald, Greifswald, Germany
2. First Clinic of Internal Medicine, Department of Internal Medicine, University of Genoa, 6 viale Benedetto XV, 16132 Genoa, Italy
3. Department of Cardiology, University Hospital Zurich, Zurich, Switzerland
4. Cardiocentro Ticino Institute, Ente Ospedaliero Cantonale (EOC), Lugano, Switzerland
5. Johnson and Johnson, Raritan, NJ, US
6. Hematology Division, Brigham and Women's Hospital, Harvard Medical School, Boston, Massachusetts, US
7. Department of Cardiology, General Hospital Klina, Brasschaat, Belgium
8. Department of Biostatistics at Epidemiology, Biostatistics and Prevention Institute, University of Zurich, Zurich, Switzerland Thrombosis Research Institute, London, UK
9. Division of Cardiovascular Medicine, Department of Medicine, Brigham and Women’s Hospital, Harvard Medical School, Boston, Massachusetts, US
10. Institute of Pharmacology, University of Bern, Bern, Switzerland
11. Department of Angiology, University Hospital Zurich, Zurich, Switzerland
12. Center for Thrombosis and Hemostasis, University Medical Center of the Johannes Gutenberg University Mainz, Mainz, Germany.

**Supplementary Figures**

**Supplementary Figure S1. PRISMA flow chart

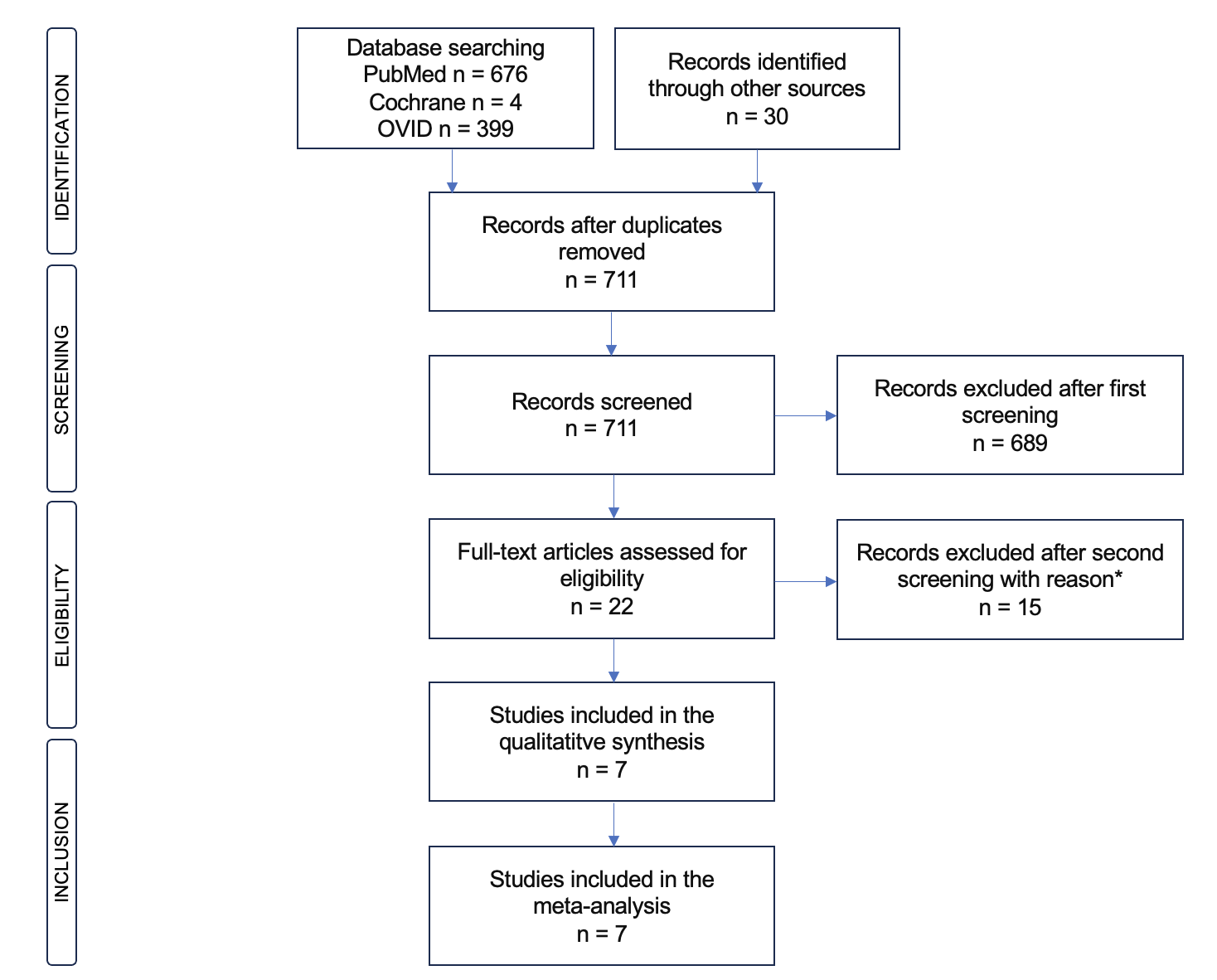
**

*

Excluded due to population: n = 4
Excluded due to type of intervention: n = 0

Excluded due to outcomes: n = 0

Excluded due to study design: n = 11

**Supplementary Figure S2. Cochrane's risk of bias assessment**

*A. Cochrane’s risk of bias assessment graph*


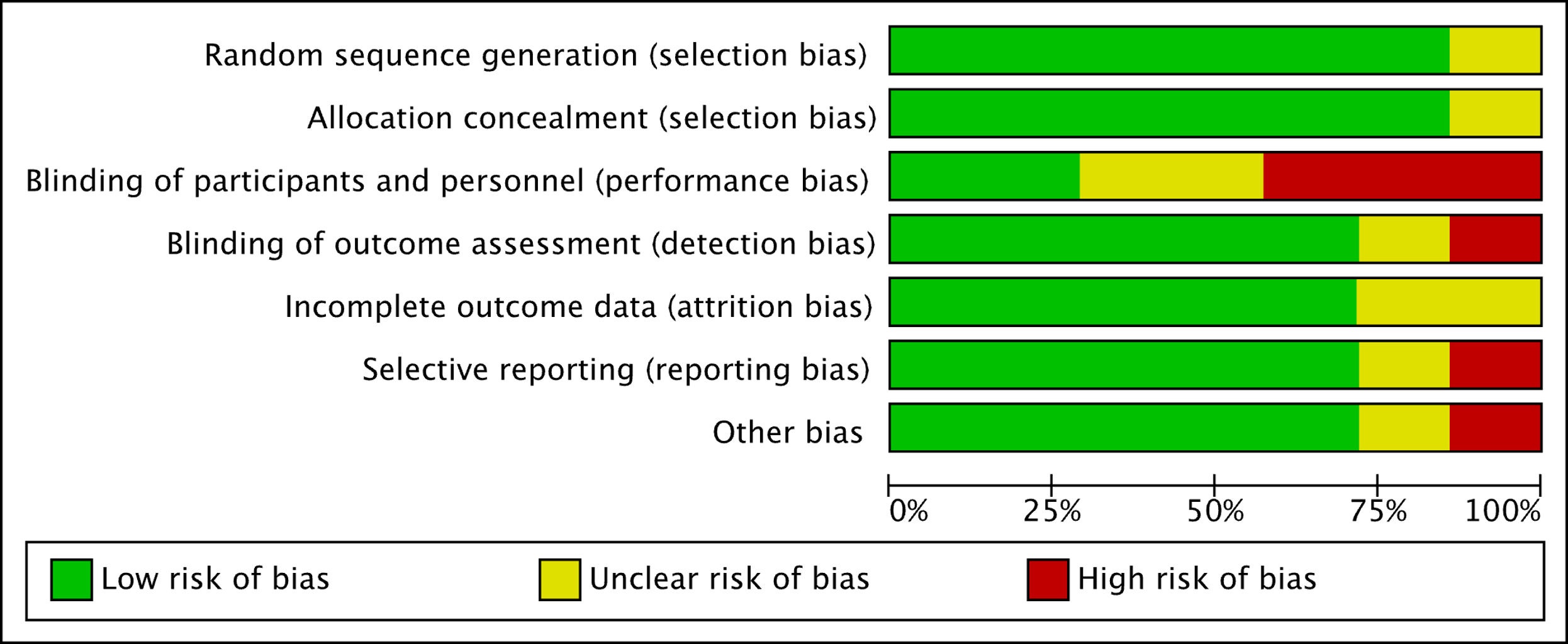


*B. Cochrane’s risk of bias assessment summary*


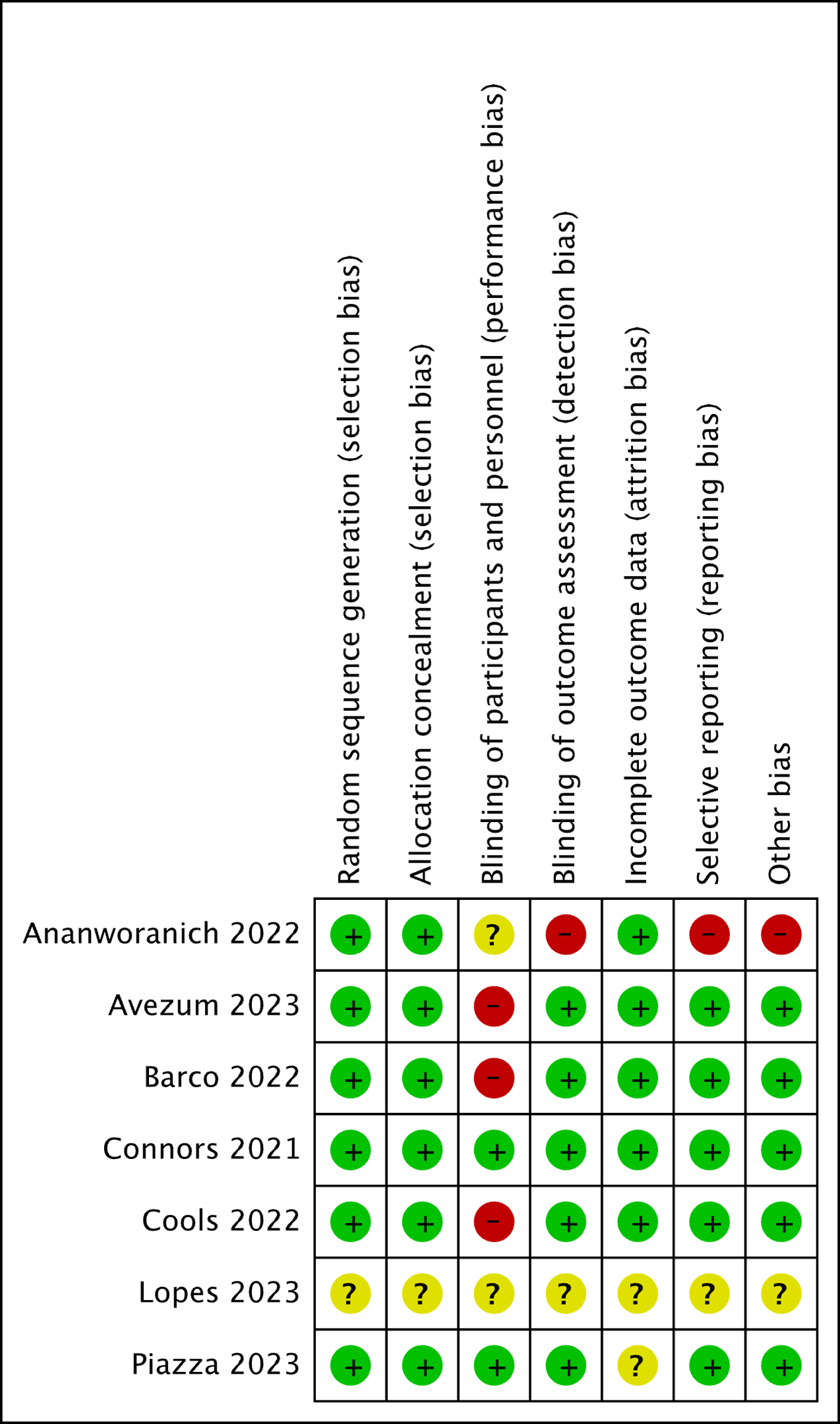


**Supplementary Figure S3.** **Secondary outcomes of the meta-analysis**

*A. Venous thromboembolism and major arterial cardiovascular events within 30 days*


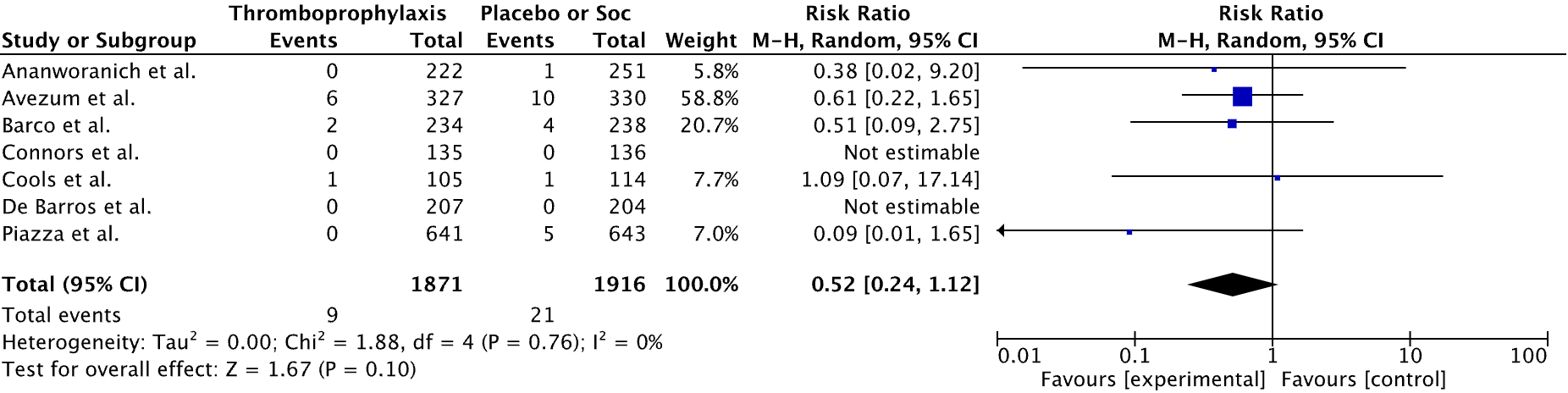


*B. All-cause hospitalisation within 30 days*


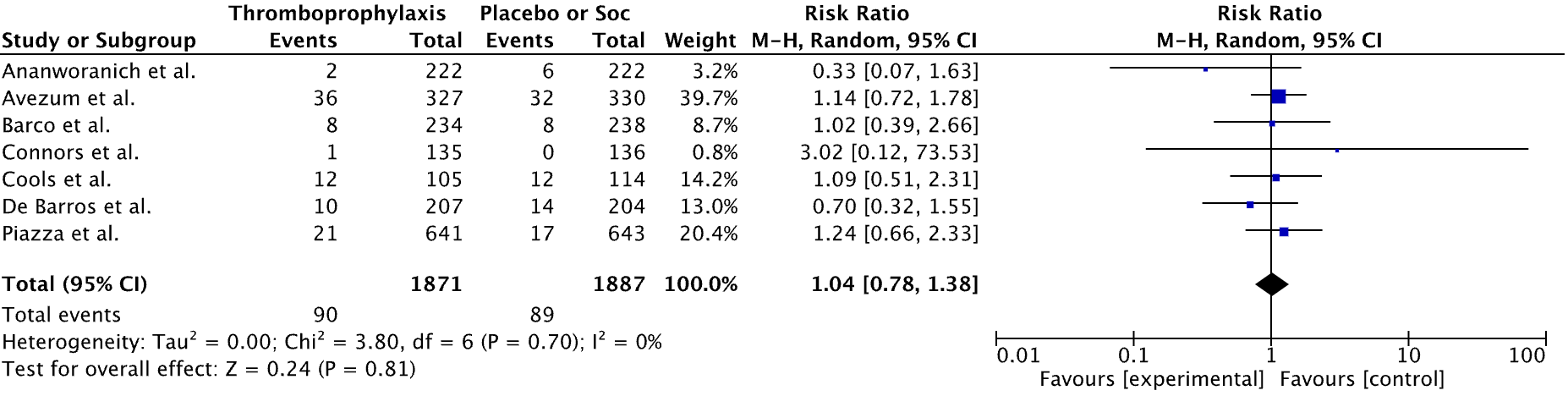


*C. All-cause death within 30 days*


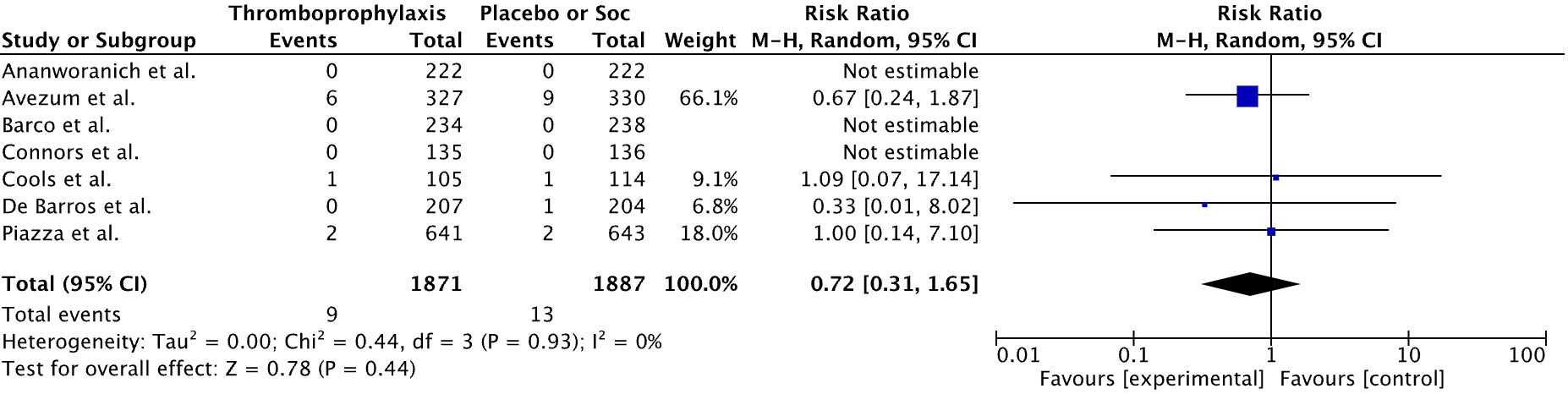


M-H, Mantel Haenszel; CI, Confidence Interval; Soc, standard of care.

**Supplementary Tables**

**Supplementary Table S1.** **Inclusion and exclusion criteria of the studies included in the meta-analysis**

| **Study** | **Inclusion Criteria** | **Exclusion Criteria** |
| --- | --- | --- |
| Connors et al. [4] | Age 40-80 years, newly diagnosed symptomatic SARS-CoV-2 infection, positive PCR or antigen test results, creatinine clearance >30 mL/min/1.73m², platelet count >100,000/mm³. | Previous hospitalization for COVID-19, acute leukemia, recent major bleeding, contraindication to or other indication for anticoagulation, need for single or dual antiplatelet therapy, pregnancy or lactating. |
| Ananworanich et al. [5] | Age ≥18, positive SARS-CoV-2 PCR test within 10 days of screening, and at least one COVID-19 symptom within 7 days of randomization. Mild COVID-19 at screening, high risk for severe COVID-19 due to age ≥65, chronic disease requiring daily treatment (diabetes, lung disease, heart disease, hypertension, cancer), or self-reported obesity. | Presence of any condition associated with an increased risk of bleeding. |
|  |  |  |
| Barco et al.[6] | Age ≥50 years, presented with acute respiratory symptoms or body temperature >37.5°C, positive test for SARS-CoV-2 within the previous 5 days. | Presence of any other condition posing an indication for anticoagulation or dual antiplatelet treatment, contraindications to anticoagulant treatment, severe renal or hepatic dysfunction, severe anemia, recent major bleeding. |
|  |  |  |
| Cools et al.[7] | Age ≥30 years, not vaccinated against COVID-19, symptomatic COVID-19 (symptoms up to 9 days; see appendix p 3 for symptoms list), confirmed by positive SARS-CoV-2 RT-quantitative PCR test, at least one risk factor for severe disease in the outpatient setting. | Contraindications to unfractionated heparin or low-molecular-weight heparin, recent (<48 hours) or planned spinal or epidural anaesthesia or puncture, percutaneous coronary intervention, thrombolytic therapy within the preceding 24 hours, increased risk of bleeding complications, pregnancy, severe renal impairment (glomerular filtration rate <30 mL/min), current anticoagulant or antiplatelet therapy (except low-dose aspirin or clopidogrel), current participation in another interventional study outside the purview of studies sponsored by the Thrombosis Research Institute, received any COVID-19 vaccines. |
|  |  |  |
| Piazza et al.[8] | Age ≥18 years, polymerase chain reaction or antigen-confirmed SARS-CoV-2 infection, symptomatic COVID-19, initial treatment plan not including hospitalization, at least one thrombosis risk factor (increased D-dimer; documented thrombophilia; previous VTE; history of cancer, coronary artery disease, peripheral artery disease, cerebrovascular disease, ischemic stroke; a predictor of adverse COVID-19 outcomes). | Positive SARS-CoV-2 antibody test at least 2 weeks after the acute infection, bleeding risk factors, therapy with prohibited medications. |
|  |  |  |
|  |  |  |
| Avezum et al.[9] | Age ≥18 years with suspected or confirmed COVID-19 of mild or moderate severity, presenting within ≤7 days from symptom onset, and at least two of the following risk factors for clinical deterioration: age >65 years, hypertension, diabetes mellitus, asthma, chronic obstructive pulmonary disease or other chronic lung disease, current smoking, immunosuppression, body mass index ≥30 Kg/m2, history of non-active cancer, bedridden patient or reduced mobility (cannot walk ≥50% of the awake time), previous history of VTE, or use of oral hormonal contraception. | Clinical indication for hospitalization, positive test for influenza at first medical care, known hypersensitivity to rivaroxaban, any known liver disease associated with coagulopathy (INR>1.5), pregnancy, lactation, persons of childbearing age not using reliable contraceptive methods, increased risk of bleeding, stroke in the last 30 days or history of hemorrhagic or lacunar stroke, any intracranial bleeding, intracranial neoplasm, brain metastasis, arteriovenous malformation or brain aneurysm, heart failure with left ventricular ejection fraction <30% or NYHA class III or IV symptoms, eGFR <30 mL/min, dual antiplatelet therapy or full anticoagulation indication, severe thrombocytopenia (platelet count <50,000/mm3), known non-cardiovascular disease associated with poor prognosis, systemic treatment with strong CYP 3A4 and p-glycoprotein inhibitors, current treatment with investigational drugs, concurrent participation in another experimental study for COVID-19, and use of chloroquine or hydroxychloroquine with azithromycin. |
|  |  |  |
| De Barros et al.[10] | Outpatients with COVID-19 confirmed by routine tests, symptoms for ≤10 days, and one of the following risk factors: D-dimer ≥2 times upper limit of normal, C-reactive protein ≥10 mg/L, or ≥2 risk factors (D-dimer level ≥upper limit of normal, C-reactive protein ≥upper limit of normal, age ≥65 years, diabetes, chronic kidney disease stage 3, cardiopulmonary disease, history of venous thromboembolism, nursing home resident or severely restricted mobility, body mass index ≥30 kg/m^2). | Formal indication for therapeutic anticoagulation, contraindication to study drug, situations of high risk of bleeding. |

PCR, polymerase chain reaction; VTI, venous thromboembolism; GFR, glomerular filtration rate.

**Supplementary Table S2.** **Period of enrolment for the studies included in the meta-analysis**

| **Study** | **Period of enrollment** |
| --- | --- |
| Connors et al.[4] | September 1, 2020 - June 17, 2021 |
|  |  |
| Ananworanich et al.[5] | August 16, 2020 - February 3, 2021 |
|  |  |
| Barco et al.[6] | August 15, 2020 - January 14, 2022 |
|  |  |
| Cools et al.[7] | October 27, 2020 - November 8, 2021 |
|  |  |
| Piazza et al.[8] | August 2020 - April 2022 |
| Avezum et al.[9] | September 29, 2020 – May 23, 2022 |
|  |  |
| De Barros et al.[10] | March 4, 2021 - March 28, 2022 |

**Literature-search strategy**

("COVID-19"[All Fields] OR "COVID-19"[MeSH Terms]) OR "sars-cov-2"[MeSH Terms] OR "sars-cov-2"[All Fields] OR "severe acute respiratory syndrome coronavirus 2"[All Fields]) AND (("anticoagulants"[Pharmacological Action] OR "anticoagulants"[MeSH Terms] OR "anticoagulan*"[All Fields] OR "anticoagulan*"[All Fields] OR “thromboprophylaxis"[All Fields] OR “thrombosis prevention"[All Fields] OR "apixaban"[Supplementary Concept] OR "apixaban"[All Fields] OR "rivaroxaban"[Supplementary Concept] OR "rivaroxaban"[All Fields] OR "dabigatran"[Supplementary Concept] OR "dabigatran"[All Fields] OR "heparin*"[Supplementary Concept] OR "heparin*"[All Fields] OR "apixaban"[Supplementary Concept] OR "apixaban"[All Fields] OR "edoxaban"[Supplementary Concept] OR "edoxaban"[All Fields]) AND ("clinical trials as topic"[MeSH Terms] OR ("clinical"[All Fields] AND "trials"[All Fields] AND "topic"[All Fields]) OR "clinical trials as topic"[All Fields] OR "trial"[All Fields] OR "trial's"[All Fields] OR "trialed"[All Fields] OR "trialing"[All Fields] OR "trials"[All Fields])

**PubMed/Medline**

676 results on 11. Dec 2023

(COVID-19 or sars-cov-2) and (anticoagulation or thromboprophylaxis or apixaban or rivaroxaban or dabigatran or heparin or edoxaban or thrombosis prevention or thromboprophylaxis)

**Cochrane Collaboration**

4 results on 11. Dec 2023.

("COVID-19" OR "severe acute respiratory syndrome coronavirus 2") AND ("outpatients") AND ("venous thromboembolism" OR "VTE") AND ("rivaroxaban" OR "enoxaparin" OR "apixaban" OR "edoxaban" OR "dabigatran") AND ("randomized controlled trial" OR "RCT") AND ("2020" OR "2021" OR "2022" OR "2023")
